# Congenital toxoplasmosis presenting as central diabetes insipidus in an infant: a case report

**DOI:** 10.1186/1756-0500-7-184

**Published:** 2014-03-28

**Authors:** Sarar Mohamed, Abdaldafae Osman, Nasir A Al Jurayyan, Abdulrahman Al Nemri, Mustafa AM Salih

**Affiliations:** 1 Department of Pediatrics (39), College of Medicine, King Saud University, P.O. Box 2925, 11461 Riyadh, Saudi Arabia

**Keywords:** Congenital toxoplasmosis, Central diabetes insipidus, Infant

## Abstract

**Background:**

Congenital toxoplasmosis has a wide range of presentation at birth varying from severe neurological features such as hydrocephalus and chorioretinitis to a well appearing baby, who may develop complications late in infancy. While neuroendocrine abnormalities associated with congenital toxoplasmosis are uncommon, isolated central diabetes insipidus is extremely rare.

**Case presentation:**

Here, we report on a female infant who presented with fever, convulsions, and polyuria. Examination revealed weight and length below the 3^rd^ centile along with signs of severe dehydration. Fundal examination showed bilateral chorioretinitis. This infant developed hypernatremia together with increased serum osmolality and decreased both urine osmolality and specific gravity consistent with central diabetes insipidus. Serology for toxoplasma specific immunoglobulin M was high for both the mother and the baby and polymerase chain reaction for toxoplasma deoxyribonucleic acid was positive in the infant confirming congenital toxoplasmosis. Brain computerized tomography scans demonstrated ventriculomegaly associated with cerebral and cortical calcifications. Fluid and electrolyte abnormalities responded to nasal vasopressin therapy.

**Conclusion:**

This report highlights central diabetes inspidus as a rare presentation of congenital toxoplasmosis.

## Background

The incidence of maternal infection with *Toxoplasma gondii* during pregnancy ranges from 1 to 8 per 1000 pregnancies [1]. The risk of vertical transmission of this obligate intracellular parasite to the fetus increases with the gestational age rising from less than 2% at 4 gestational weeks to more than 80% at 36 weeks of pregnancy [1-4].

Congenital toxoplasmosis (CTox) is usually asymptomatic. However, acute manifestations in the neonatal period may occur including fever, anemia, jaundice, seizure and hydrocephalus [5]. Few infants with CTox may present with late sequel such as senso-neural deafness, microcephaly and developmental delay. Infants with CTox are usually treated with sulphadiazine, pyrimethamine, and folinic acid for three months. Treatment may be extended to one year depending on the severity of the disease and the response to therapy [6].

Involvement of hypothalamic-pituitary axis is rarely reported with CTox [7-11]. This usually affects the anterior pituitary and exceptionally the posterior pituitary. We here report on an infant with congenital toxoplasmosis who presented with central diabetes insipidus (DI) without other endocrine abnormalities involving the hypothalamic pituitary axis.

## Case presentation

A term baby girl was born to a 37-year-old Saudi woman, with a birth weight of 2600 grams. The mother had fever, body aches, and macular skin rash for a few days in the 3^rd^ trimester. Antenatal scan was normal, and antenatal serology screening for congenital infections was not done. Six days after birth, the baby was admitted with fever and convulsions, She was diagnosed as neonatal sepsis, however; septic screening was unremarkable including a negative blood culture. Similar sepsis like episodes with fever and convulsions recurred at 3 and 6 weeks of life, which were treated with intravenous antibiotics, and the baby was discharged home in a good condition. At the age of 3 months, the diagnosis of DI was suspected when she presented with significant polyuria (urine output was around 15 ml/kg/hr), severe dehydration, and convulsion. Although systemic examination was normal at birth, she started to show up abnormal neurological features by 3 months of age including: bilateral nystagmus, significant head lag, hypertonia, brisk reflexes, and extensor planter response, along with failure to thrive. Examinations of the fundus showed bilateral chorioretinitis.

**Figure 1:**
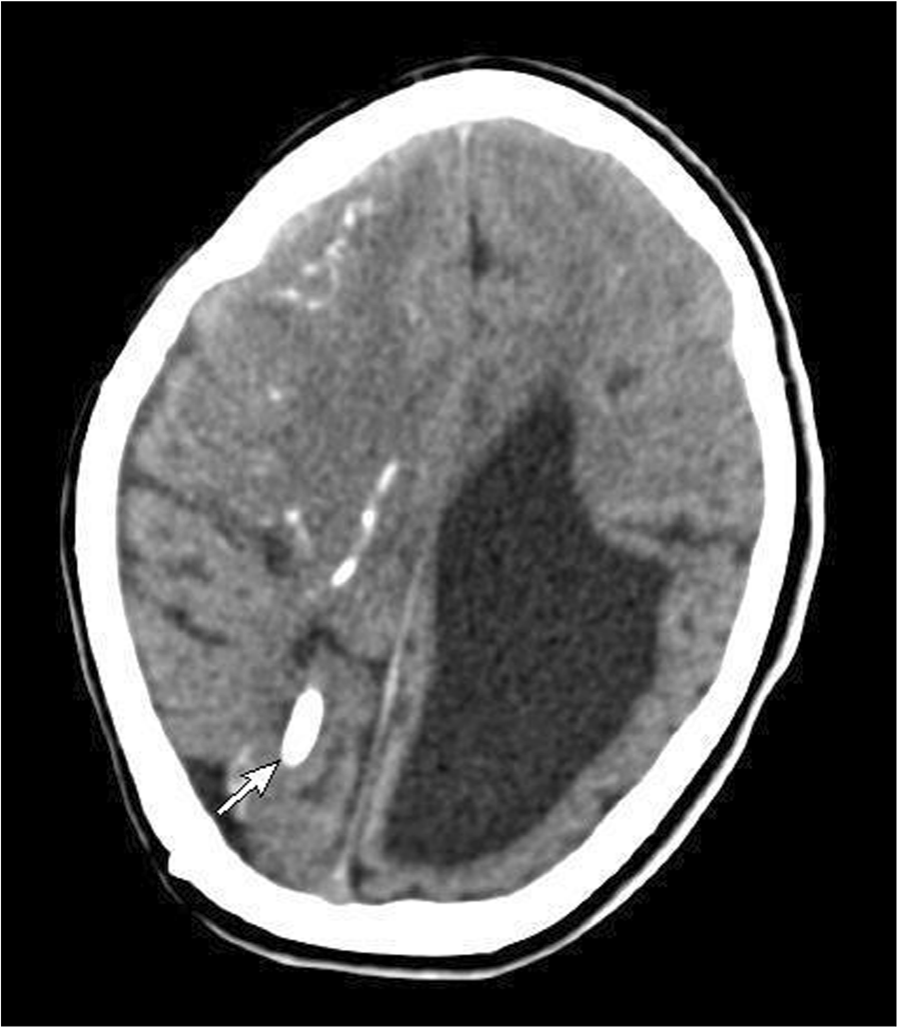
Computerized tomography of brain showing dilated ventricles with multiple subependymal and parenchymal calcifications (arrow).

Renal function showed normal urea and creatinine, however; serum sodium was ranging between 147 and 150 mmol/L. Urine was diluted with urine osmolality between 100 to 110 mosm/L (normal >600 mmol/L) and she had low urine specific gravity of 1.005 (normal > 1.010). Thyroid function, growth hormone, luteinizing hormone (LH), follicle stimulating hormone (FSH), adrenocorticotrophic hormone (ACTH) and cortisol were within the normal limits (Table 1). Computerized tomography (CT) of the brain demonstrated dilated ventricles with multiple subependymal and parenchymal calcifications (Figure 1). Cerebrospinal (CSF) analysis showed high protein and cells (Table 1). Electroencephalography (EEG) revealed multifocal epileptiform discharges on diffusely slow background. Electroretinography (ERG) illustrated evidence of retinopathy on the right eye.

**Table 1:** Result of laboratory investigations for the infant with congenital toxoplasmosis at 3 months of age

**Lab work**	**Result**	**Reference value**
WBC	5.6 × 10.e9/L	4.5-13.5
HGB	85 g/L	115-145
PLT	182 × 10.e9/L	140-450
%NEUT	36.1%	40-55
%LYMP	61.8%	38-42
%MONO	1.6%	3-9
%EOS	0.2%	0-6
%BASO	0.3%	0-1
CSF WBC	14	< 5
Neutrophils	5%	
Lymphocytes	95%	
CSF protein	1240 mg/dl	25-40
CSF glucose	42 mg/dl	40-60
ACTH test	22.6 pg/mL	0-60
Cortisol AM	315 nmol/L	140-690
FT4	13.66 pmol/L	10.3-25.8
TSH	5.70 mIU/L	0.25-5
Alkaline Phosphatase	107 U/L	<250
Alanine Aminotransferase	52 U/L	10-65
Aspartate Aminotransfer	23 U/L	10-31
Gamma Glutamyl Transpep.	99 U/L	5-55
Total Bilirubin	4 UMOL/L	2-17
Direct Bilirubin	0 UMOL/L	0

WBC: White cell count; HGB: Hemoglobin; PLT: Platelet; CSF: Cerebrospinal fluid; ACTH: Adrenocorticotrophic hormone; FT4: Free thyroxine 4; TSH: Thyroid stimulating hormone.

Serology for toxoplasma specific immunoglobulin M (IgM), performed for this infant at 3 month of age using enzyme-linked immunosorbent assay (ELISA), was a 1370 unit/L (positive 120 (unit/L), and immunoglobulin G (IgG) was a 11128 unit/L (positive > 110 unit/L). As antenatal serology screening for congenital infection was not done, maternal serology for toxoplasma IgM and IgG was performed 3 month after delivery when CTox was suspected. This result showed that maternal toxoplasma specific IgM was a 630 (positive 120 (unit/L), and IgG was a 4320 unit/L (positive > 110 unit/L). Polymerase chain reaction (PCR) was positive for toxoplasma deoxyribonucleic acid (DNA) in both CSF and blood of the infant. The baby was commenced on pyrimethamine/sulphadiazine and leucovorin at 4 month of age aiming to continue this treatment for 1 year. A favorable response has been shown by regression of retinal lesions after few weeks of starting antimicrobial treatment. Also, she showed good response to nasal vasopressin with urine output reduced to 3.2 ml/kg/hr, urine osmolality increasing to 650 mosml/l and sodium controlled between 135 and 140 mmol/l. Monthly follow up routine blood tests including complete blood count (CBC), liver and renal functions showed satisfactory response to treatment and absence of side effects of antimicrobial therapy.

At age of 7 months and while still on pyrimethamine/sulphadiazine and leucovorin, the baby developed vesicular rash, high-grade fever, and convulsions. CBC was normal and CSF examination revealed protein of 1120 mg/dl, glucose of 65 mg/dl and 20 cells, mostly lymphocytes. CSF culture for bacteria as well as CSF polymerase chain reaction for *Toxoplasma gondii* was negative. Direct immunoflorescence test of a smear taken from the vesicular rash was positive for varicella. Based on this clinical data, in addition to having recent contact with a family member with active chicken pox, our patient was diagnosed as severe varicella meningoencephalitis. Compared to the previous images, CT brain in this acute stage showed that hydrocephalus was not progressive and hence ventriculoperitoneal shunt was not inserted. This patient underwent extensive immune work up including serum immunoglobulin, complement and neutrophil function that failed to identify evidence of immunodeficiency. She passed through a stormy course, became comatose with intractable seizures, irreversible shock and severe metabolic acidosis. She died despite full cardiorespiratory support with fluid, inotropes, hydrocortisone and mechanical ventilation.

## Discussion

Congenital toxoplasmosis (CTox) results from the trans-placental passage of the *Toxoplasma godii* parasite from the mother to the fetus. The majority of babies born to mothers infected by this parasite are asymptomatic. However, some babies present with variable clinical manifestations including anemia, jaundice, hepatosplenomegaly, seizures, hydrocephalus, chorioretinitis and senso-neural deafness. The severity of fetal damage depend on the stage of pregnancy when maternal infection occurs. Although she looked apparently normal at birth, our patient presented in the neonatal period with nonspecific sepsis-like picture. Later she showed predominant involvement of the central nervous system with seizures, hydrocephalus, chorioretinitis and spasticity. This clinical features alerted us to the possibility of CTox, in which the detection of specific IgM or IgA antibodies that cannot cross the placental barrier, is a key marker of fetal infection. Moreover, prenatal diagnosis relies mostly on the PCR based detection of parasite DNA. Unfortunately, this diagnostic approach was not possible in this case as antenatal screening for congenital infection is not routine practice in the hospital where our patient was born. In the absence of this antenatal serology data we performed anti-*T gondii* IgG and IgM by enzyme-linked immunosorbent assay (ELISA), which was proved to be positive for both the infant and the mother three months after delivery. This was an indirect evidence of CTox, which was ultimately confirmed by positive PCR for Toxoplasma in the infant. It is interesting that IgG was elevated in both the infant and the mother. It is expected that IgG synthesis reaches a plateau within 2 or 3 months of the acute infection and then decreases more or less rapidly and persists lifelong at residual titers, which are highly variable among patients [1]. As maternal IgG is passively transferred in utero, therefore, a qualitative analysis is required to differentiate between maternal antibodies and others synthesized by the infected infant [1]. This was not needed in our patient as IgM was positive that indicates infection.

Our patient presented with recurrent fever that can be explained by dysfunction of the hypothalamic thermoregulatory center secondary to toxoplasma infection. Fever could also result from dehydration caused by early deficiency of antidiuretic hormone that was confirmed later in the course of the disease. However, the serum electrolyte pattern in the first episodes of fever was not consistent with diabetes insipidus. Serum urea is usually elevated in dehydrated children secondary to poor feeding or fluid loss resulting from diarrhea and or vomiting. Interestingly, serum urea is usually normal in dehydrated patients with diabetes insipidus as in our patient [12]. This results from defective reabsorption of urea in the distal renal convoluted tubules that is normally facilitated by antidiuretic hormone. This useful clinical clue should raise the suspicion of diabetes inspidus in any child with unexplained dehydration and normal serum urea.

CTox may affect the hypothalamic pituitary axis resulting in variable deficiencies of the anterior as well as the posterior pituitary hormones. Isolated and selective deficiency of the antidiuretic hormone as in our patient is unusual. Massa *et al*. [8] reported three cases of CTox and hypothalamic-pituitary dysfunction, all of whom presented with different patterns of combined hormonal deficiency (GH deficiency, diabetes insipidus, tertiary hypothyroidism, adrenal insufficiency, and delayed puberty). Siahanidou *et al*. [13] described a newborn similar to our patient, who presented with prolonged fever and was confirmed later to have central DI, hypothyroidism, and ACTH deficiency. Few cases with isolated DI as the only endocrine abnormality associated with CTox were described in the literature. Karadag *et al*. [14] reported a neonate with CTox and hydrocephalus, who developed DI at the 10^th^ day of life. Similarly, Oygur *et al*. [15] presented a 33-day-old boy who had polyuria, hypernatremia, and dehydration secondary to central DI associated with CTox.

Although the diagnosis of DI was suspected late, our patient responded nicely to nasal vasopressin with fluid and electrolytes reverting to normal shortly after starting replacement therapy [16]. Later, she had a stormy acute course with most likely varicella meningoencephalitis that led to her death. This working diagnosis is supported by the typical rash, documented contact with a relative with varicella, elevated CSF protein and cells, in addition to the positive direct immunoflorescence test for varicella. However, this diagnosis could have been confirmed beyond doubt should varicella PCR has been performed. Other possible explanation of this severe disease was entertained such as immunodeficiency and adrenal insufficiency, but none of these possibilities was proven despite extensive workup. Nevertheless, our patient was empirically covered with stress dose of intravenous hydrocortisone during her acute illness. The possibility of antimicrobial drug adverse effects was considered, however; this event occurred 3 months after starting treatment. Moreover, the rash was typical of varicella infection.

## Conclusion

Antenatal and newborn screening for CTox offers early diagnosis and treatment of affected mother and infant with specific antimicrobial therapy that leads to a better outcome. This case report with severe manifestations of CTox and delayed diagnosis of the disease highlights the importance of antenatal and newborn screening for toxoplasmosis, which is a feasible measure to minimize the complications of CTox [1,4,5,17]. This report also alerts physicians to central diabetes inspidus as a rare presentation of CTox.

## Consent

Written informed consent was obtained from the parents of the infant for publication of this Case Report and any accompanying images. A copy of the written consent is available for review by the Editor-in-Chief of this journal.

## Abbreviations

CTox: Congenital toxoplasmosis; DI: Diabetes inspidus; CT: Computerized tomography; ACTH: Adrenocorticotrophic hormone; FSH: Follicle stimulating hormone; LH: Luteinizing hormone; GH: Growth hormone; TSH: Thyroid stimulating hormone; ELISA: Enzyme-linked immunosorbent assay; IgA: Immunoglobulin A; IgG: Immunoglobulin G; IgM: Immunoglobulin M.

## Competing interests

The authors declare that they have no competing interests.

## Authors' contributions

SM designed the paper, collected the data, reviewed the literature, drafted and reviewed the manuscript. AO, NAA, AA, MAMS, participated in data collection, writing and reviewing the manuscript. All authors read and approved the final manuscript.

## Authors’ information

Sarar Mohamed (SM) is Fellow of the Royal College of Pediatrics and Child Health. He is Consultant Pediatric Endocrinologist and Associate Professor of Pediatrics at King Saud University, Riyadh, Saudi Arabia. Abdaldafae Osman (AO) is Assistant Pediatric Consultant at King Saud University. Nasir A Al Jurayyan (NAA) MD is Professor of Pediatrics and Consultant Pediatric Endocrinologist at King Saud University. Abdulrahman Al Nemri (AA) is Associate Professor of Pediatrics and Consultant Endocrinologist at King Saud University. Mustafa AM. Salih (MAMS) is Fellow of the Royal College of Pediatrics and Child Health, Professor of Pediatrics, and Consultant Pediatric Neurologist, College of Medicine, King Saud University, Riyadh, Saudi Arabia.
